# Delirium Associated with Salicylate and Acetaminophen Overdose in a Patient with COVID-19: A Case Report

**DOI:** 10.5811/cpcem.2020.8.48559

**Published:** 2020-09-08

**Authors:** Tyler H. Wen, Jason Chu, Danielle M. Allenspach, David C. Van

**Affiliations:** * Columbia University Vagelos College of Physicians and Surgeons, New York, New York; † Columbia University Irving Medical Center, Department of Emergency Medicine, New York, New York

**Keywords:** COVID-19, delirium, neuropsychiatric effects, overdose

## Abstract

**Introduction:**

The coronavirus disease 2019 (COVID-19) pandemic has created numerous clinical challenges for physicians, in part due to its wide range of clinical manifestations and associated complications.

**Case Report:**

Here we present the case of a 69-year-old man who was admitted to the emergency department with fever, dyspnea, and altered mental status. We believe the patient’s condition was precipitated by a COVID-19 infection-induced delirium, a setting in which he ingested aspirin and acetaminophen in overdose that required lifesaving interventions.

**Conclusion:**

This case illustrates the potential for neuropsychiatric effects in COVID-19 patients due to both direct viral central nervous system pathology and pandemic-related psychosocial stressors.

## INTRODUCTION

During the coronavirus disease 2019 (COVID-19) pandemic, our New York City hospital emergency department (ED) was inundated with patients who presented with respiratory symptoms typical of the COVID-19 infection (dyspnea, fever, and dry cough).^1^ While many patients develop subsequent renal, cardiovascular, or other organ complications, psychiatric or mental status changes are less common.^1^,^2^ We report a case of a 69-year-old patient with COVID-19 symptoms with concurrent altered mental status (AMS). Although initial workup revealed active COVID-19 infection, the workup of AMS represented a diagnostic challenge.

## CASE REPORT

A 69-year-old male with diabetes mellitus and hypertension was brought to the ED during the height of the COVID-19 outbreak with fever, dyspnea, and AMS. The patient’s agitation and confusion stymied interview attempts, but a health records review showed he had been evaluated in the ED three days earlier for fever and cough. He was thought to have a probable COVID-19 infection at that time, but his condition did not warrant admission and the ED was not offering COVID-19 testing for outpatients.

Initial vital signs were notable for tachycardia to 120 beats per minute, blood pressure of 140/90 millimeters of mercury (mm Hg), tachypnea to 22 breaths per minute, oxygen saturation of 96% on room air, and an oral temperature of 38.2º Celsius. Staff noted his breaths were remarkably deep with coarse basilar lung sounds. The patient was in moderate respiratory distress with diaphoresis, cold and mottled extremities and 5-millimeter pupils. No focal neurological deficits or signs of head trauma were noted, but the patient was acutely agitated and restless, shifting repeatedly in the stretcher from side to side.

The providers contacted his wife by telephone and learned that his medications included acetaminophen and insulin. She related that after returning home from the ED three days earlier, he began self-treating his fever but appeared increasingly confused. His wife denied that the patient had any psychiatric or substance abuse history.

A differential diagnosis at the time included infectious etiologies (e.g., sepsis syndrome, encephalitis); drug toxicity or withdrawal; neurological events (e.g., stroke); and metabolic disorders (e.g., hyperglycemic hyperosmolar state, hypoglycemia). His initial workup revealed a bedside fingerstick glucose of 234 milligrams (mg) per deciliter (dL) (reference range [ref]: 70–99 mg/dL), benign electrocardiogram and head computed tomography findings, and bilateral opacities on chest radiograph consistent with viral pneumonia. Severe acute respiratory syndrome coronavirus 2 (SARS-CoV-2) reverse transcriptase-polymerase chain reaction test was positive. Laboratory tests were as follows: arterial blood gas- pH 7.50 (ref: 7.35–7.45); partial pressure of carbon dioxide 18 mm Hg (ref: 35–45 mm Hg); partial pressure of oxygen 98 mm Hg (ref: 80–100 mm Hg); bicarbonate 14 millimoles (mmol) per liter (L) (ref: 22–29 mmol/L); serum anion gap 27 (ref: 8–17); aspartate aminotransferase 69 units (U) per L (ref: 5–40 U/L); and alanine aminotransferase 51 U/L (ref: 7–56 U/L). The blood gas and metabolic panel revealed a mixed anion gap metabolic acidosis with respiratory alkalosis.

With this new information, clinical staff pivoted to focus on toxic and metabolic etiologies. A serum toxicology screen showed a salicylate level of 52.2 mg/dL (therapeutic range: 0–30mg/dL); acetaminophen level of 70.8 microgram (mcg) per mL (therapeutic range: 10–30 mcg/mL); and undetectable ethanol level. Poison control was consulted, and the patient was admitted to the intensive care unit for urinary alkalization with a sodium bicarbonate infusion for salicylate toxicity and intravenous N-acetylcysteine for acetaminophen toxicity. However, he became oliguric with persistently elevated salicylate levels and refractory confusion requiring hemodialysis. The patient’s mental status improved over three days after hemodialysis.

Upon regaining mental clarity, the patient volunteered that he had deliberately taken large quantities of aspirin and acetaminophen in an attempt to take his own life. He expressed feeling helpless after believing that he had an untreatable COVID-19 infection. His worsening dyspnea further exacerbated the mental anguish, leading him to become increasingly certain that he was dying. The patient also became more confused in the days leading up to his admission, and stated he felt he had no choice but to take large amounts of pain medication.

Eventually he endorsed both lapses and confabulations in his memory during the peak of his symptoms, and thought that his confusion and impaired judgment precipitated the medication overdose. He denied any current suicidal ideation. Psychiatry consultants agreed that the overdose primarily occurred in the context of delirium and felt that his risk of future self-harm was low since he had no history of suicidality or underlying psychiatric disorder. The patient was ultimately released 10 days after admission with community support referrals but no new medications.

CPC-EM Capsule

What do we already know about this clinical entity?

*While the respiratory, cardiovascular and renal effects of coronavirus disease 2019 (COVID-19) are well described, understanding of its brain effects is limited and evolving*.

What makes this presentation of disease reportable?

*There is a growing realization that COVID-19 can have neuropsychiatric effects, but reports in the medical literature are limited*.

What is the major learning point?

*COVID-19, along with advanced age, fever, societal stressors and pharmacologic effects, can all contribute to neuropsychiatric dysfunction in affected patients*.

How might this improve emergency medicine practice?

*Our case highlights an infrequently encountered condition for emergency practitioners to consider when managing altered mental status in patients with COVID-19*.

## DISCUSSION

In retrospect, the patient’s presentation of fever and AMS with a mixed anion gap metabolic acidosis and respiratory alkalosis was consistent with salicylate toxicity, a condition whose pathophysiology and treatment protocols are well established.^3^,^4^ Unique to this case was its situation within the COVID-19 pandemic, which was a distracting alternate etiology for the patient’s fever. This was bolstered by his prior ED visit which, although testing was not indicated, was consistent with a COVID-19 infection. At that time, the patient was discharged with acetaminophen due to reassuring vital signs; his mental status only began to deteriorate after returning home.

While acute salicylate poisoning may have contributed to the patient’s presentation of AMS, neurological findings are typically associated with higher levels of serum salicylate concentrations (80–90 mg/dL) than those observed in our patient (52 mg/dL).^5^ Alternatively, chronic salicylate toxicity can manifest with a variety of neurologic findings (including confusion, delirium, agitation, and coma) associated with lower serum salicylate levels.^5^ In our case the ingestion pattern remains unclear: the patient was unsure whether he had taken a bottle of 100 “PM” pills and acetaminophen as a single ingestion or as multiple large doses over several days. Regardless, a history of confusion preceding the overdose suggests that a COVID-19 infection was independently responsible for his initial delirium.

Present data suggests that the SARS-CoV-2 virus responsible for COVID-19 may induce an over-exaggerated host immune response (“cytokine storm”) causing increased systemic vascular permeability and a state of viral sepsis with multiorgan dysfunction.^1^,^2^ This increase in endothelial permeability along the blood-brain barrier (BBB) can lead to cerebral edema and subsequent central nervous system (CNS) dysfunction.^2^ Furthermore, there is evidence suggesting that SARS-CoV-2 can invade the CNS; viral ribonucleic acid has been detected in the CNS neuronal cells of COVID-19 patients, and autopsies have revealed cerebral hyperemia with neuronal degeneration.^6^,^7^ Proposed mechanisms of CNS viral entry include viral invasion of peripheral nerves with retrograde spread and trans-synaptic transmission, transcribial spread via olfactory epithelial cells expressing angiotensin-converting enzyme 2 receptors, direct viral invasion of vascular endothelial cells along the BBB, and viral infection of leukocytes that migrate through the BBB (Trojan horse mechanism).^6^,^8^

There has also been at least one reported case of COVID-19-associated acute necrotizing encephalopathy, a rare complication of viral infections that results from neurotoxic cytokine accumulation and cerebral edema-induced necrosis.^2^,^9^ AMS can be an indicator of overall COVID-19 illness severity. Mao et al found that 15% of inpatients with severe COVID-19 infections presented with impaired consciousness during the early days of the pandemic in Wuhan, China, compared to 7.5% of inpatients with non-severe courses.^6^

Apart from physiological factors that increase risk of delirium (including direct viral CNS pathology, pharmacological effects, hypoxemia, fever, and advanced age), we believe that the COVID-19 pandemic’s widespread social impacts also contributed to the patient’s psychiatric decompensation and suicidality.^10^ Our patient related pre-admission feelings of despair and hopelessness regarding his respiratory symptoms, perceiving that he had an untreatable and fatal COVID-19 infection. This milieu of despair was possibly compounded by physical isolation, media coverage of high mortality rates, a lack of definitive treatments, and a pervasive sense of uncertainty.^11^,^12^ Social isolation hindered access to community venues for discussing his fears, further exacerbating his mental anguish (Figure).

Li et al showed an increase risk of suicide in patients with mental disorders and socioeconomic deprivation, which may be heightened in a pandemic.^13^ Suicides directly attributed to a fear of COVID-19 infection and isolation measures have already been reported.^14^ Lastly, the unique burden of managing hospitalized patients in the “COVID era” has also affected healthcare staff, in whom rates of psychological stress, burnout and depression appears to have risen.^15^ Clearly the increased workload in some centers is a factor, but many clinicians have managed COVID-19-infected colleagues, experienced uncertainty of personal protective equipment availability, and needed to provide unprecedented personal attention to patients whose family members were disallowed from visiting at the bedside. All these factors predicate the need for increased awareness of the current pandemic’s profound psychosocial impact on both patient and provider.

## CONCLUSION

Our case suggests an increased risk of altered mental status in COVID-19 patients due to a combination of neurophysiological and psychosocial stressors. If unrecognized and untreated, patients with delirium can potentially exhibit dangerous behaviors such as self-harm or suicidal ideation; our patient was driven to an overdose of multiple medications as a sequela of his delirium and required lifesaving interventions. Informing families of COVID-19 patients to stay vigilant for signs of confusion or AMS may help avert similar episodes. Additional close monitoring and proactive management of cognitive changes should be considered for high-risk patients, such as those living alone or lacking social support.

## Figures and Tables

**Figure:**
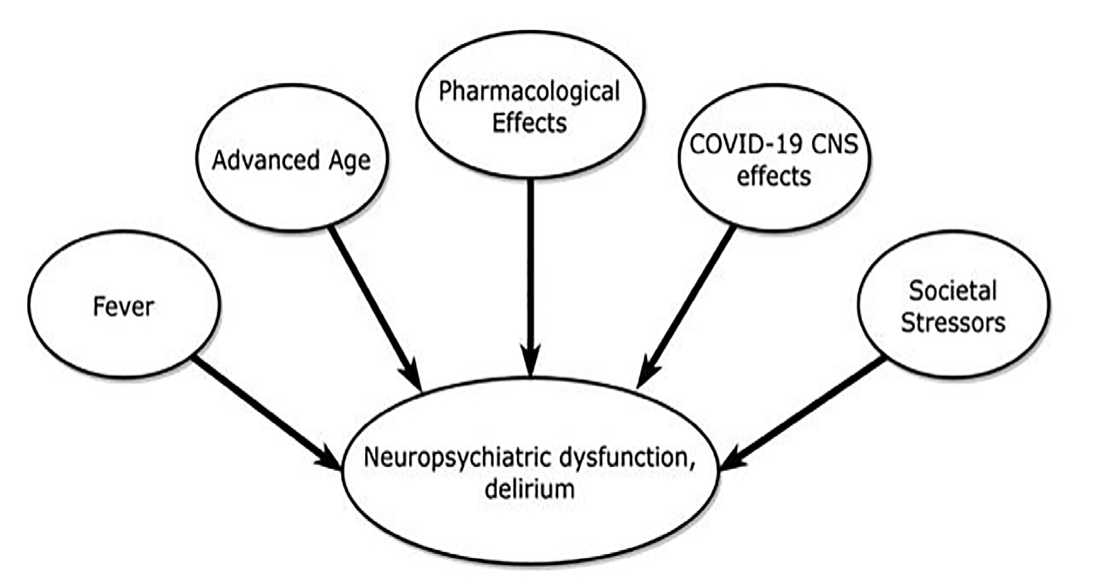
Numerous factors are likely to contribute to delirium in patients with coronavirus disease 2019 infection. *COVID-19*, coronavirus disease 2019; *CNS*, central nervous system.
